# Extracranial arteriovenous malformations: a 10-year experience at a German vascular anomaly center and evaluation of diagnostic imaging for endovascular therapy assessment

**DOI:** 10.3389/fmed.2024.1473685

**Published:** 2024-12-02

**Authors:** Nadja Werba, Johannes Ludwig, Christel Weiss, Felix Struebing, Stefan Schoenberg, Maliha Sadick

**Affiliations:** ^1^Clinic for Radiology and Nuclear Medicine, University Medical Centre Mannheim, Mannheim, Germany; ^2^Department of Medical Statistics, Biomathematics and Information Processing, Medical Faculty Mannheim, Ruprecht-Karls-University of Heidelberg, Heidelberg, Germany; ^3^Department of Hand, Plastic and Reconstructive Surgery, Burn Center, Berufsgenossenschaft (BG) Trauma Centre, Ludwigshafen, Germany

**Keywords:** vascular anomalies, arteriovenous malformation (AVM), diagnostic imaging analysis, magnetic resonance imaging (MRI), computed tomography

## Abstract

**Background:**

Arteriovenous malformations (AVMs) account for <3% of vascular anomalies. This study aims to present the 10-year experience of a German vascular anomaly center (VAC) with AVMs and evaluate diagnostic imaging for treatment-relevant information for minimally invasive therapy planning.

**Material and methods:**

A retrospective study including patients from the VAC database with AVMs was conducted. Clinical information from patients' records was evaluated. An additional image reading analysis of the available diagnostic imaging using a 4-point Likert scale, focusing on relevant points for minimally invasive treatment planning, was conducted in 13 patients who had all three magnetic resonance tomography (MRI), computed tomography (CT), and conventional angiography available.

**Results:**

Between April 2014 and March 2024, 60 patients (60% female, 40% male; 12% Parkes Weber syndrome) with AVMs presented to the VAC. The median age was 36 years (range: 11–78 years). Referral diagnosis was correct in 73.3% of cases. The mean distance to the VAC was 102.5 km (±111.0). The most common locations involved the hand (32%), lower extremity (22%), and pelvis (22%). The most common symptoms were pain (81%), pulsation (64%), and local hyperthermia (62%). Necrosis was significantly more common when the AVM was located in the hand (*p* = 0.0129) and growth when located in the pelvis (*p* = 0.0037). Furthermore, cosmetic issues were significantly more frequent when the AVM was located in the head area (*p* = 0.0333). Most patients presented with Schobinger stage II (57%). Right heart strain was only documented in one case. A total of 47% had undergone invasive therapies before VAC admission. In 30% of cases, further minimally invasive or invasive therapy was required. In the diagnostic imaging evaluation, conventional angiography had the overall best ratings for image quality (median = 1.00; range: 1.00–2.00), NIDUS evaluation median = 1.00; range: 1.00–2.00), and therapy planning (median = 1.00; range: 1.00–1.33).

**Conclusion:**

Our 10-year experience showed that in patients with AVMs, the correct diagnosis is often made before admission to a specialized VAC. Diagnostic imaging is essential for endovascular treatment planning, with conventional angiography showing superior utility in image quality, NIDUS evaluation, and therapy planning compared to other modalities.

## Introduction

Arteriovenous malformations (AVMs) are a subtype of vascular malformations, accounting for < 3% of vascular malformations, and are characterized by their fast-flow dynamics due to a direct connection between high-pressure arteries and low-pressure veins (nidus), inducing extensive shunting and shunt related symptoms (1–3). They can occur in simple forms or in combination with other anomalies, such as Parkes-Weber syndrome, which is defined by the presence of an AVM with multiple microfistulae, capillary malformation, and limb overgrowth (1).^1^ They are already present at birth but grow and likely progress with the patient's age (4). Puberty, trauma, and pregnancy in women may be associated with exacerbation risk (1, 5–7). Clinically, they can be categorized using Schobinger stages. Local cutaneous blush and warmth define Stage I. Bruit, pulsation, and growth of the AVM can be seen in Stage II, while pain, ulceration or necrosis, and bleeding define Stage III. Stage IV is characterized by decompensation and heart failure (5) (Table 1). Diagnostics are based on clinical features and imaging tools, including ultrasound (US), especially color Doppler examination, magnetic resonance imaging (MRI), computed tomography (CT), and digital subtraction angiography (DSA) (8, 9). Because of treatment complexity, only symptomatic AVMs with Schobinger stages III and IV, or stage II if well-localized, should be considered for non-conservative treatment (5). Conservative treatment options vary depending on the location of the AVM and include watch-and-wait, compression garments, and pain medication. Minimally invasive and invasive treatment indications should always be discussed in an interdisciplinary team (10) and treatment path should be chosen depending on the stage of the AVM and the patient's symptoms.

**Table 1:** Schobinger classification of arteriovenous malformations.

Stage I	Quiescence	Cutaneous blush, skin warmth, arteriovenous shunt on Doppler ultrasound
Stage II	Expansion	Pulsation, thrill and bruit, expanding lesion
Stage III	Destruction	Pain, dystrophic skin changes, ulceration, bleeding
Stage IV	Decompensation	Cardiac failure

Occlusion of the NIDUS is the mainstay of treatment and therefore the evaluation of flow and shunt dynamics and configuration of the NIDUS are essential information provided by diagnostic imaging.

This study aims to share a 10-year experience of a German vascular anomaly center (VAC) in the management of AVMs and evaluate imaging modalities for appropriate assessment of required minimally invasive therapy.

## Methods

We conducted a retrospective single-center study including patients with an arteriovenous malformation to evaluate the clinical presentation, imaging differences as well as therapy indication, and outcome from a 10-year-experience period database of a German VAC. Information regarding demographics, referral, and therapy for all vascular anomalies in this VAC, including 49 AVMs, from 2014 to 2021 has been published elsewhere (11). However, the research question addressed in our current study differs from the one mentioned, and therefore also the specific analyses. We also obtained ethical approval from the local ethics committee. Patients with arteriovenous malformations who presented to the VAC since its initiation in April 2014 to March 2024 were included. Data were collected from electronic medical records and from information provided by the patient regarding their medical history during the VAC consultation. Furthermore, four available imaging modalities (US, MRI, CT, and DSA) were individually documented for each patient.

According to their age at first presentation to the VAC, patients were categorized into the following age groups: infants (0–12 months), toddlers (1–4 years), children (5–13 years), juveniles (14–17 years), and adults (over 18 years). The straight-line distance from patients' residences to the VAC, along with the correlation between referral diagnoses and final diagnoses, was evaluated. Information on referring clinics and medical specialization was collected. Localization of the AVM was categorized into the following anatomical regions: hand, upper extremity, foot, lower extremity, pelvis, abdomen, chest, neck, and extracranial head region. Intracranial and cerebral AVMs are entirely managed by the Department of Neuroradiology and Clinic for Neurosurgery at our university hospital, not by the VAC; therefore, we had to explicitly exclude them. At the time of initial consultation, symptoms were evaluated using the recorded information, and Schobinger classification (5) was complemented with the documented information on symptoms and clinical appearance in the VAC reports. Pain intensity was documented using a 0–10 numeric scale for adults with a minimum of 0 and a maximum of 10, and a visual analog scale for children. Diagnostics and therapies before and after the VAC, as well as the outcome at last contact, were evaluated.

Evaluation of imaging criteria was performed in cases where all three modalities (CT, MRI, and transarterial angiography) were available for each patient. The availability of ultrasound was not an inclusion criterion. Imaging modalities were independently analyzed by a senior radiologist with over 15 years of experience in vascular anomalies (reader 1), a radiologist without specific knowledge of vascular anomalies (reader 2), and a radiology resident with at least 3 years of experience in vascular anomalies (reader 3). Furthermore, all readers had different experience levels in interventional radiology. The following aspects using a 4-point Likert scale (1 = well-assessable, 2 = moderately well-assessable, 3 = poorly assessable, and 4 = not assessable or no images available) were evaluated:

- Image quality regarding the AVM
- Assessability of the AVM nidus
- Assessability of tissue involvement and precise AVM localization
- Assessability of AVM volume
- Image information value for minimally invasive treatment planning

### Statistical analysis

All statistical calculations were performed using SAS software, release 9.4 (SAS Institute Inc., Cary, North Carolina, USA). For quantitative variables, mean values and ranges were calculated. For qualitative factors, absolute and relative frequencies were given.

Fisher's exact test was applied to test the association between two binary factors. To compare multiple imaging modalities based on readers' assessments, several Kruskal–Wallis tests were conducted. If the result of a Kruskal–Wallis test was statistically significant, pairwise comparisons were performed using the Wilcoxon two-sample tests. Because of the rather small sample sizes, Bonferroni correction was not applied. In general, test results with *p*-values < 0.05 were considered statistically significant.

## Results

### Demographics

From April 2014 to March 2024, a total of 905 patients presented to the VAC. Sixty patients were diagnosed with an arteriovenous malformation, of which seven (12%) patients had Parkes-Weber syndrome. Thirty-six (60%) were female patients and 24 (40%) were male patients. Median age was 36 years (range: 11–78 years) with 53 (88%) adults, 3 (5%) juveniles, and 4 (7%) children. There were no infants or toddlers diagnosed with a congenital arteriovenous malformation. Referral diagnosis was correct in 44 (73%) patients, and 9 (15%) patients presented with incorrect diagnosis. Most referrals were from external clinics (20 patients, 34%), with vascular surgeons being the primary referrers (nine patients, 39%). The main on-campus referrer was dermatology (five patients, 46%). Sixteen (27%) patients were self-admitted without direct referral. Eleven (19%) patients had only one physician's appointment regarding their vascular malformation prior to consultation at the VAC. The median distance from the place of patients' residence to the VAC was 102.5 km (±111.0), with 28 (47%) patients living in the same state, 25 (42%) in the neighboring states, and 7 (12%) in other states of Germany (Table 2).

**Table 2:** Demographic characteristics.

***n* = 60**
**Sex**	
Male	24 (40%)
Female	36 (60%)
**Age**	
0–12 months	0
1–4 years	0
5–13 years	4 (6.7%)
14–17 years	3 (5%)
>18 years	53 (88.3%)
**Residence**	
Same state	28 (46.7%)
Surrounding states	25 (41.6%)
Other states	7 (11.7%)
**Referral**	
In house	11 (18.3%)
Ex domo clinic	20 (33.3%)
Practice	12 (20%)
Self-admission	16 (26.7%)
Not documented	1 (1.7%)

### Spectrum of symptoms

**Figure 1:**
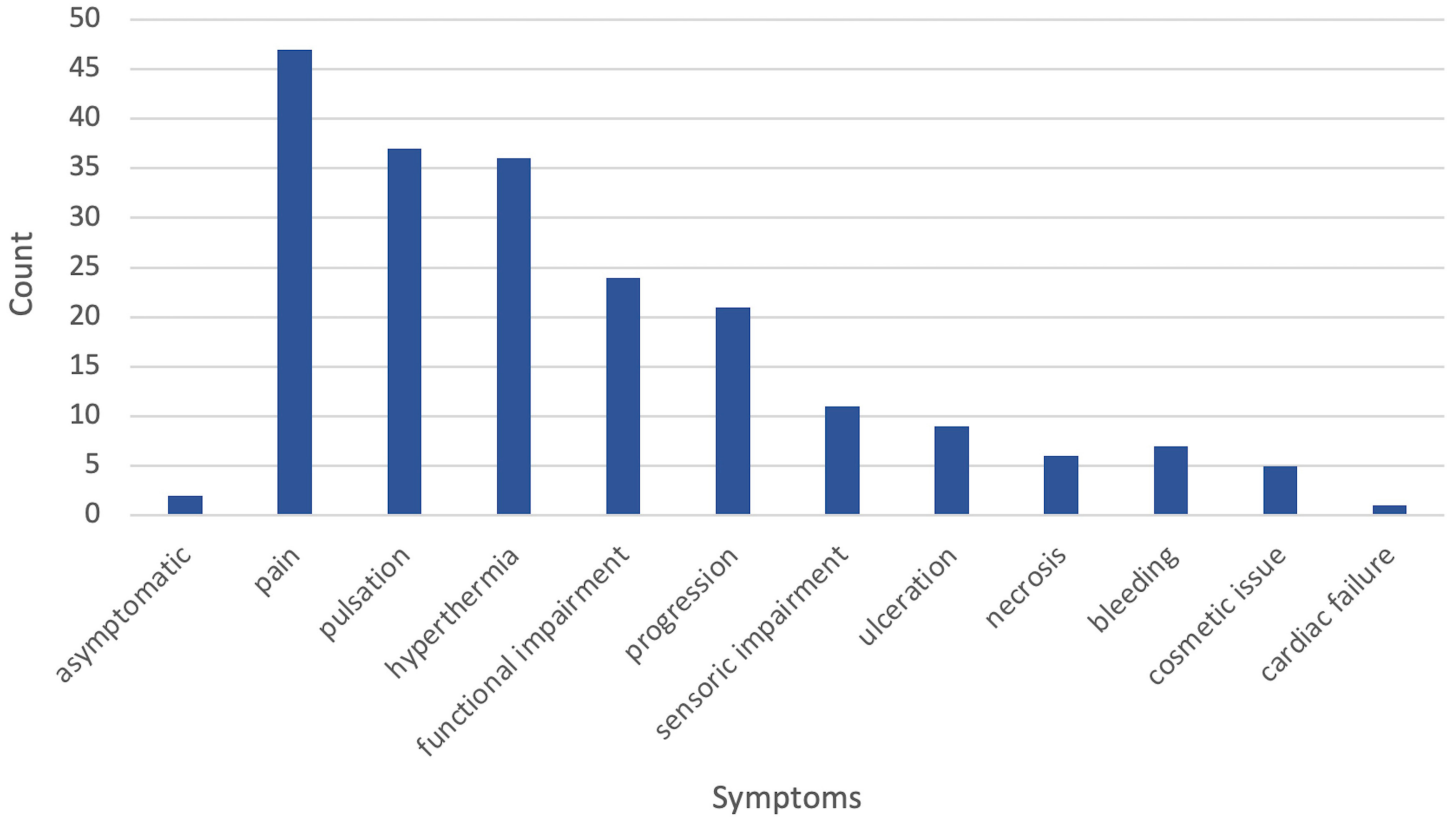
Frequency of documented clinical symptoms at initial presentation to our vascular anomaly center (VAC).

Symptoms at the time of initial presentation to the VAC were recorded. Only two patients (3%) were asymptomatic. Regarding the clinical presentation, most patients presented with Schobinger stage II (34 patients, 57%) and stage III (10 patients, 17%), whereas stage I (six patients, 10%), I–II (two patients, 3%), II–III (three patients, 5%), III–IV (three patients, 5%), and stage IV (two patients, 3%) were rare. Where overlapping stages were documented in the available records, these were adopted as valid. In symptomatic patients, the following symptoms were recorded: the most common symptom overall was pain in 47 (81%) patients, followed by pulsation (37 patients, 64%) and local hyperthermia (36 patients, 62%). Functional impairment and progression of the AVM were also very common, with 42% (24 patients) and 40% (21 patients), respectively. Eleven patients (19%) reported sensory impairment. Ulceration and tissue necrosis were recorded less often, only in nine (15%) and six (10%) patients, respectively. Furthermore, local bleeding of the AVM was rare (seven patients, 12%). An explicit cosmetic issue was documented in five (9%) patients. There was only one recorded case (2%) of cardiac failure associated with the AVM. Figure 1 gives an overview of clinical symptoms. Further reported symptoms in one individual case after previous upper limb amputation were motoric dysfunction and recurring headaches due to asymmetrical weight distribution.

**Figure 2:**
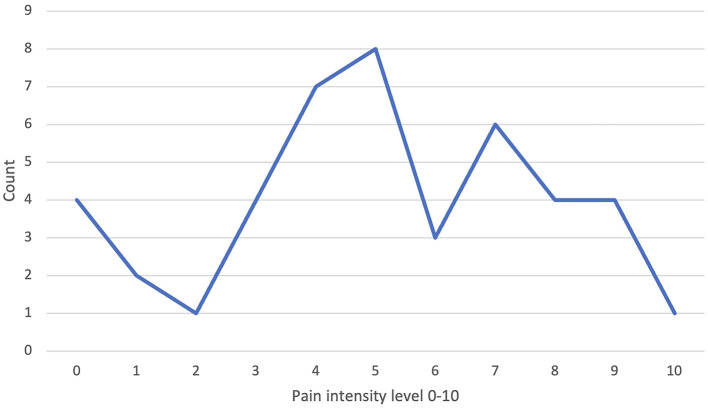
Documented pain intensity at initial consultation in our vascular anomaly center (VAC) on a numeric pain scale.

Forty-four patients specified their pain intensity at the time of first consultation on a numeric pain scale, with the majority indicating a pain intensity of five and four on a scale with a maximum of ten (13 and 12%, respectively) (Figure 2).

We conducted a subgroup analysis to compare symptom frequency based on AVM localization, with a particular focus on motoric impairment. Although there was no significant difference in the frequency of motoric impairment in comparison to the AVM location (*p* = 0.4776), we observed significantly higher rates of necrosis in patients with AVMs located on the hand (*p* = 0.0129) and recorded growth for AVMs located in the pelvis (*p* = 0.0037). Furthermore, patients who primarily reported cosmetic issues were significantly more likely to have AVMs located in the head area (*p* = 0.0333).

We also evaluated differences in symptoms comparing AVMs and Parkes-Weber syndrome. Statistical analyses showed no differences in the frequency of pain or pain intensity, local hyperthermia, motoric or sensory impairment, bleeding incidences, ulcerations or necrosis, or cosmetic issues. The only significant difference was the frequency of pulsation (*p* = 0.0003), where no documented pulsation was found in patients with Parkes-Weber syndrome. Furthermore, none of the patients with Parkes-Weber syndrome had documented right heart insufficiency or heart failure.

### Malformation characteristics

**Figure 3:**
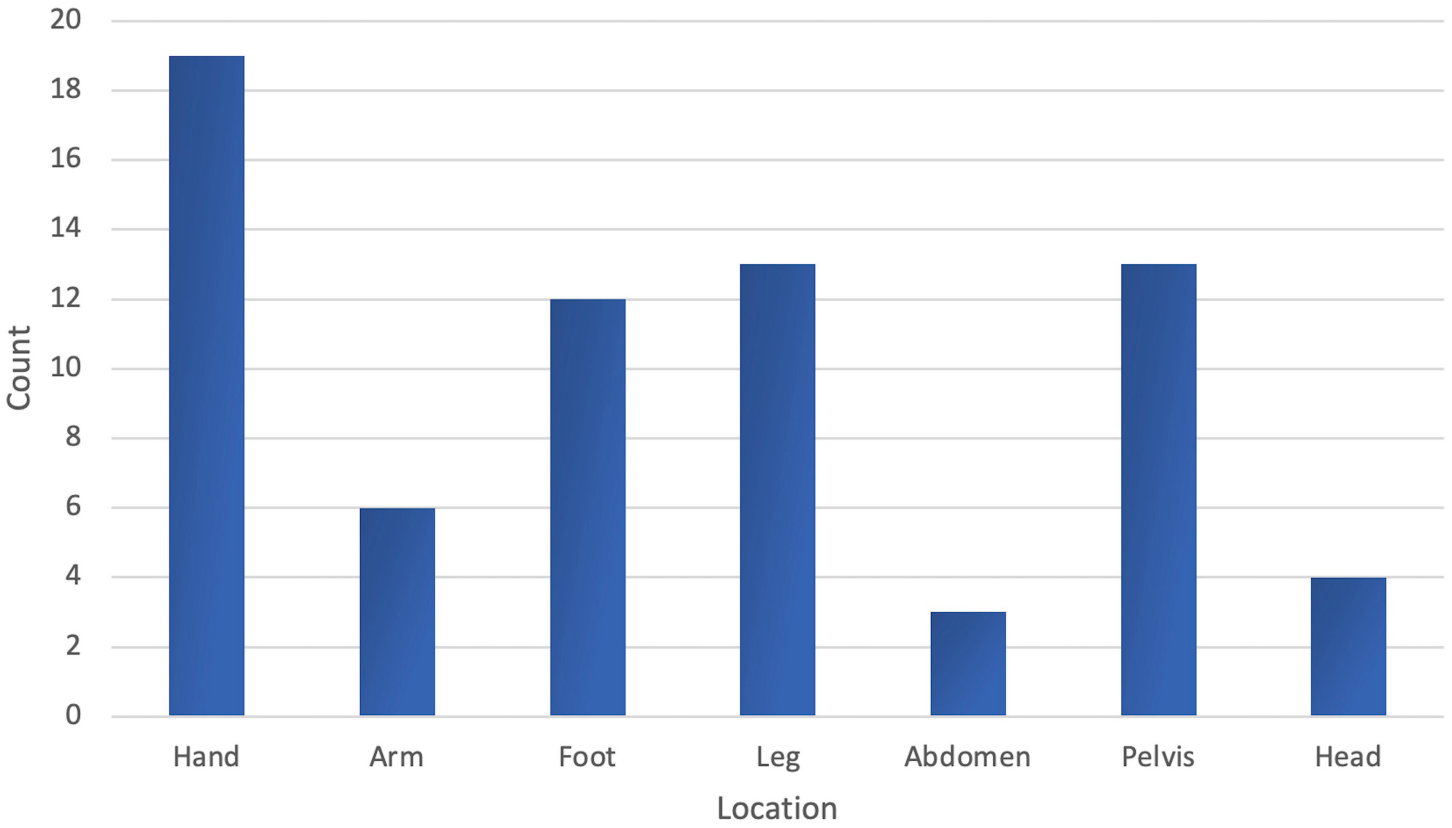
Frequency of the anatomical regions involved by the arteriovenous malformation.

The AVM was mostly located in the hand affecting 19 patients (32%). This was followed by the lower extremities and pelvis, each involving 13 patients (22% each). The foot was affected in 12 patients (20%), the upper extremities in six patients (10%), the head (extracerebral) in four patients (7%), and the abdomen in three patients (5%). There were no AVMs located on the chest or neck (Figure 3). In 57 patients (95%), the AVMs only involved one location; segment-overlapping locations were rare.

### Diagnostics

**Figure 4:**
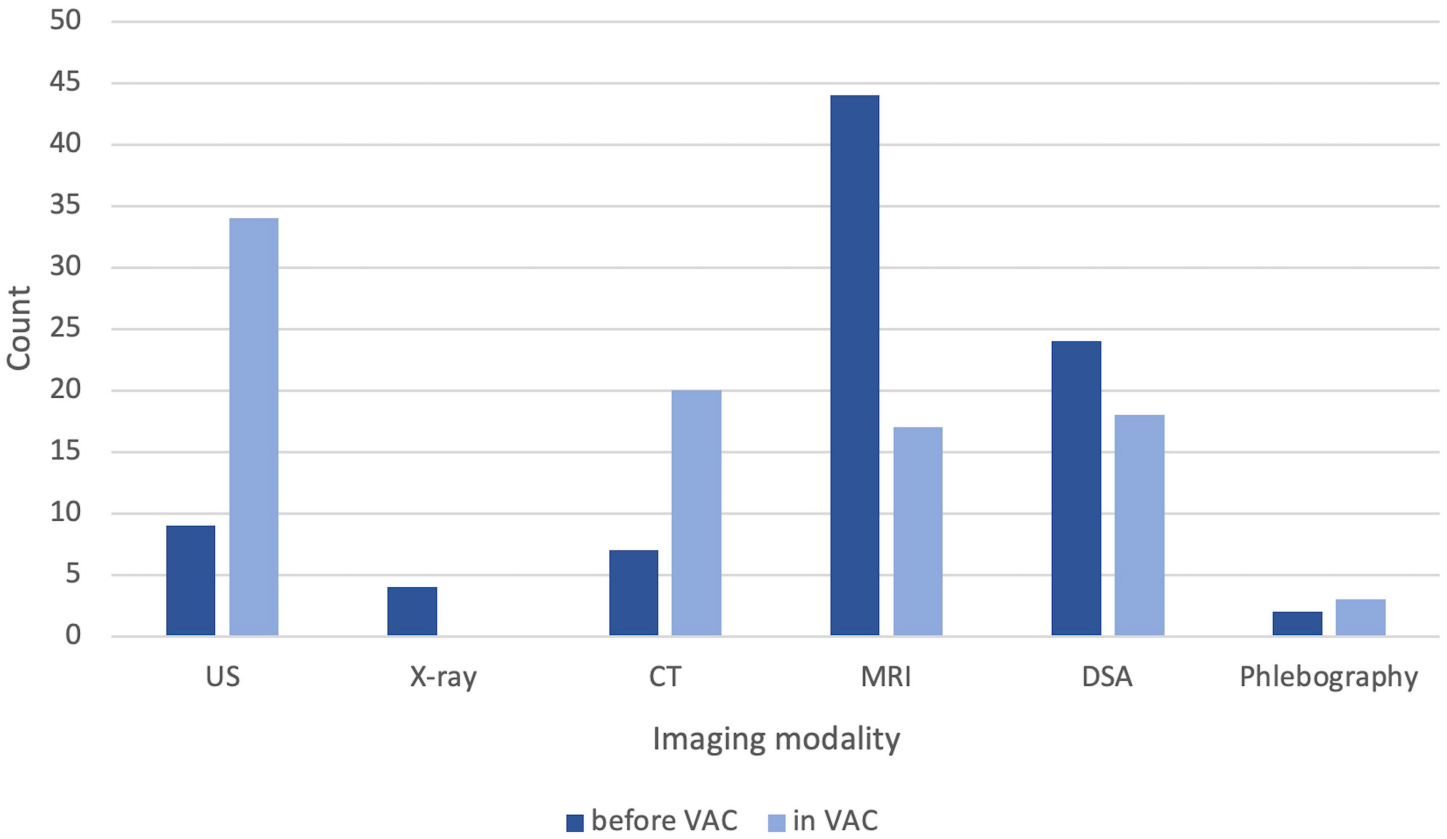
Documented diagnostic imaging per patient before and after admission to our vascular anomaly center (VAC). US, ultrasound; CT, computed tomography; MRI, magnetic resonance imaging; DSA, digital subtraction angiography.

A median of 1 (range: 0–4) imaging modalities were already performed before admission to the VAC. 44 (73%) patients had undergone MRI imaging, 24 (40%) transarterial angiography, followed by 9 (15%) confirmable ultrasounds, 7 (12%) CT scans, 4 (7%) conventional x-rays, and 2 (3%) phlebographies. In 28 (47%) cases, imaging was sufficient. In the VAC, we performed ultrasound in 34 (57%) patients, CT in 20 (33%), MRI in 17 (28%) patients, and diagnostic transarterial angiographies in 18 (30%) patients. Conventional phlebography was performed significantly less frequently, with only three (5%) documented cases (Figure 4). The median number of imaging modalities in the VAC was 1 (range: 1–5).

### Comparison of diagnostic imaging modalities

Out of a total of 60 patients, 13 (22%) underwent all three imaging modalities (MRI, CT, and DSA), and the examinations were available in our imaging database. Of these 13 patients, six patients also had at least 1 US to evaluate. Average reader assessment of the four imaging modalities showed a generally poor rating for the US in all evaluated aspects. In the category of image quality, DSA showed the best average rating (median = 1.00; range 1.00–2.00). In the category of tissue involvement, MRI (median = 1.33; range 1.00–2.33) and CT (median = 1.33; range 1.00–2.00) were similarly well-rated. For NIDUS evaluation (median = 1.00; range 1.00–2.00) and therapy planning (median = 1.00; range 1.00–1.33), DSA received the best rating. For volume assessment, MRI received the best rating (median = 1.33; range 1.00–3.00), closely followed by CT (median = 1.67; range 1.00–2.33) and DSA (median = 1.67; range 1.00–3.00). When the US is included in the statistical analysis for reader assessment, significant differences are observed in all categories due to the uniformly poor rating of the US in the overall reader assessment. After excluding the US and only comparing MRI, CT, and DSA, significant differences in the reader's assessment could still be shown in the categories for tissue involvement (*p* < 0.0001), NIDUS evaluation (*p* = 0.0003), and therapy planning (*p* < 0.0001).

For each of these three categories, pairwise comparisons were performed. Rating for tissue involvement showed a significant difference when comparing DSA to MRI (*p* < 0.0001) and CT (*p* < 0.0001) independently, but not when comparing MRI and CT (*p* = 0.6316). Similar results were shown for NIDUS evaluation: DSA vs, MRI (*p* = 0.0002), DSA vs, CT (*p* = 0.0126), and MRI vs, CT (*p* = 0.0505). Furthermore, for therapy planning: DSA vs, MRI (*p* < 0.0001), DSA vs. CT (*p* = 0.0201), but with a significant difference comparing MRI to CT (*p* = 0.0030), with a better rating for CT.

### Therapies

Before admission to the VAC, 28 (47%) patients underwent invasive therapy, of which eight patients underwent more than one therapeutic method: 16 surgeries (27%), 14 (23%) transarterial embolizations, 4 (7%) percutaneous sclerotherapies, one laser therapy, and one transarterial stent implantation. Ten patients (17%) were provided with compression garments, and in three (5%) cases, oral anticoagulation was prescribed.

**Figure 5:**
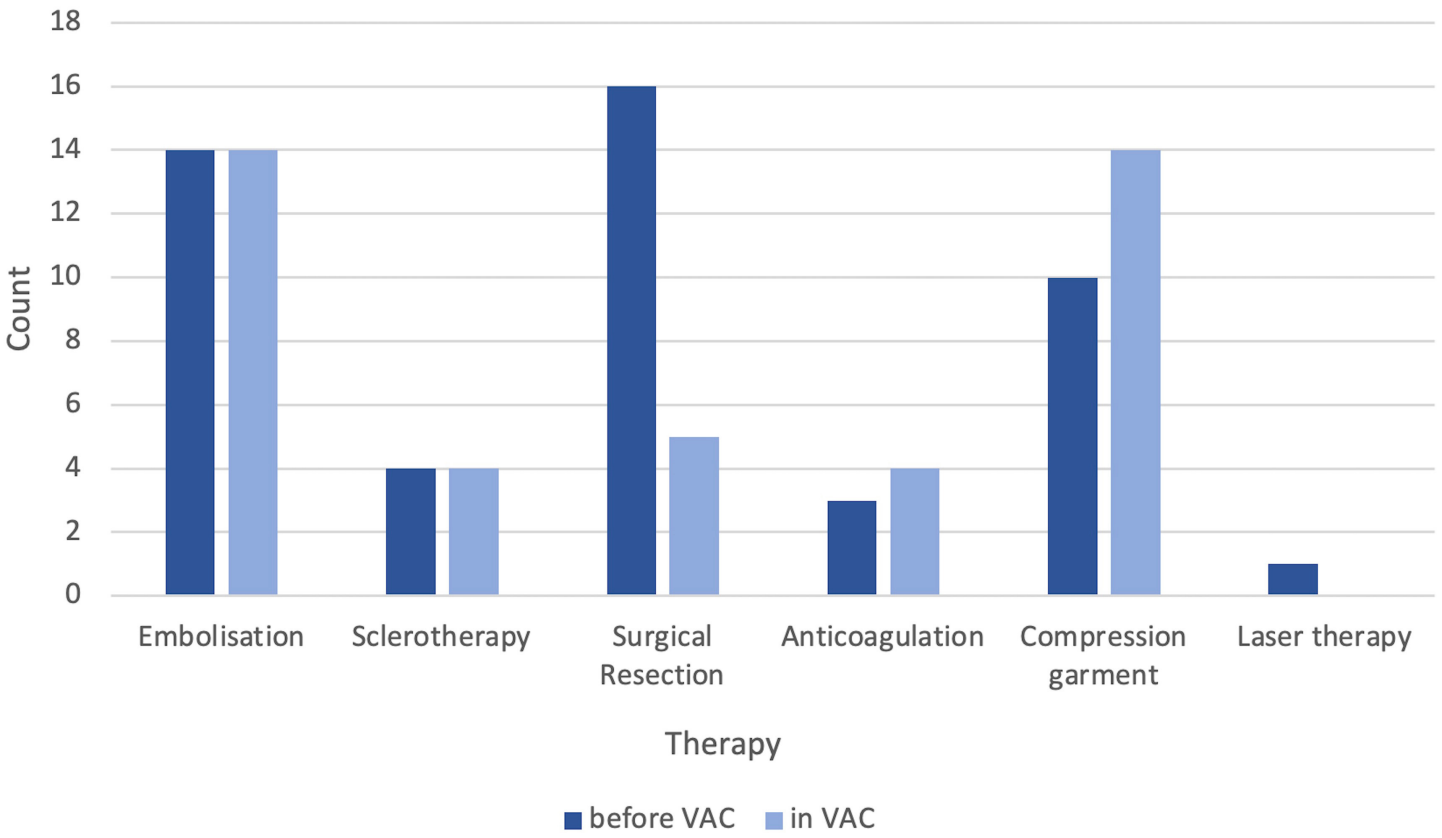
Documented therapies (conservative, minimally invasive, and invasive) before and after admission to our vascular anomaly center (VAC).

After admission to the VAC, 18 (30%) patients indicated invasive or minimally invasive therapy. A total of 14 (23%) transarterial embolizations were performed, with three (5%) cases receiving complementary percutaneous sclerotherapy and one case (2%) receiving only percutaneous sclerotherapy in a patient with Parkes-Weber syndrome. Postinterventional surgical resection was performed in three (21%) out of 14 patients, with only one patient requiring primary resection without prior transcatheter embolization. Conservative therapies, including compression garments and oral anticoagulation, were indicated in 14 (23%) and 4 (12%) cases, respectively (Figure 5). Overall, 1–3 embolization sessions per patient were performed in the VAC.

Subgroup analyses showed no significant difference in frequency comparing non-invasive vs. invasive or minimally invasive in regard to Schobinger classification or AVM localization.

After minimally invasive or surgical interventions in the VAC, 7 (39%) periinterventional complications occurred: 3 (43%) cases of delayed wound healing, 2 (29%) cases of nerve irritation, 1 (14%) case of non-target embolization, and 1 (14%) case of non-treatment-related complications. Overall, the complication rate was 39%. The median number of per-person appointments in the VAC was 2 (range: 1–8). At the time of the last consultation, 15 (25%) patients were free of symptoms with or without invasive/minimal-invasive therapy, 20 (33%) patients were categorized as “consistency of symptoms” or “symptom progression”, and 17 (28%) patients were lost to follow-up after only one appointment.

## Discussion

The aim of this study was to present a 10-year experience of a German VAC with AVMs and to evaluate the diagnostic imaging for treatment-relevant information.

Patients with AVMs presenting at our VAC were, on average, older than patients with other vascular malformations (12). This correlates with other studies showing that AVMs tend to be treated later in life, mostly during adolescence or adulthood (13–15). Furthermore, there is a slight predominance of female patients, which is similar to other studies (13, 15–17); however, no sex predilection has been reported for sporadic AVMs (7). The referral diagnosis was accurate in 73% of cases (44 patients), which contrasts with a collective that includes all vascular malformations. Strubing et al. report a correct referral rate of only 44% (11). In a study by Greene et al., correct referral diagnosis for vascular malformations overall was 46%. Arteriovenous malformations have been diagnosed correctly in 59%, venous malformations only in in 31% (18). This may be due to the fact that AVMs with their fast-flow characteristics, especially on Doppler ultrasound, are easier to diagnose than slow-flow malformations.

The most reported symptoms in our patients were pain, pulsation, and local hyperthermia, whereas bleeding, ulceration, or necrosis was less common, and especially right heart failure was seldom (Figure 1). Motoric impairment in our patients was often due to pain or difference in extremity length. On the other hand, patients with Parkes-Weber syndrome in our cohort did not report or show pulsation at the AVM location in comparison to classic AVMs. In our patient group, there was a significantly higher rate of necrosis for hand AVMs. Furthermore, growth of the AVM when located in the pelvis and cosmetic issues related to AVMs in the extracranial head area were observed significantly more often.

Comparing the AVM localization to other studies, our cohort showed a similar distribution pattern in extracranial AVMs, with the upper and lower extremities being involved more often than the abdomen and chest (Figure 3) (15).

Surgical resection may be considered in localized and well-defined AVMs (10, 19, 20), where resection with or without prior embolization has shown a lower recurrence rate (19). Transarterial embolization can be performed preoperatively to reduce blood loss in large or diffuse AVMs, in those with involvement of vital structures, or in those not feasible for resection (10, 19, 21). In our cohort, 70% of patients (*n* = 42) were managed conservatively. In those with indications for minimally invasive or invasive therapy, 14 (23%) patients underwent embolization, with three of these patients subsequently undergoing surgical cast resection. Only one patient underwent primary surgery. The overall complication rate was comparable to other studies (22–24), although it varies depending on factors such as embolic agent, AVM localization, and extent (20).

**Figure 6:**
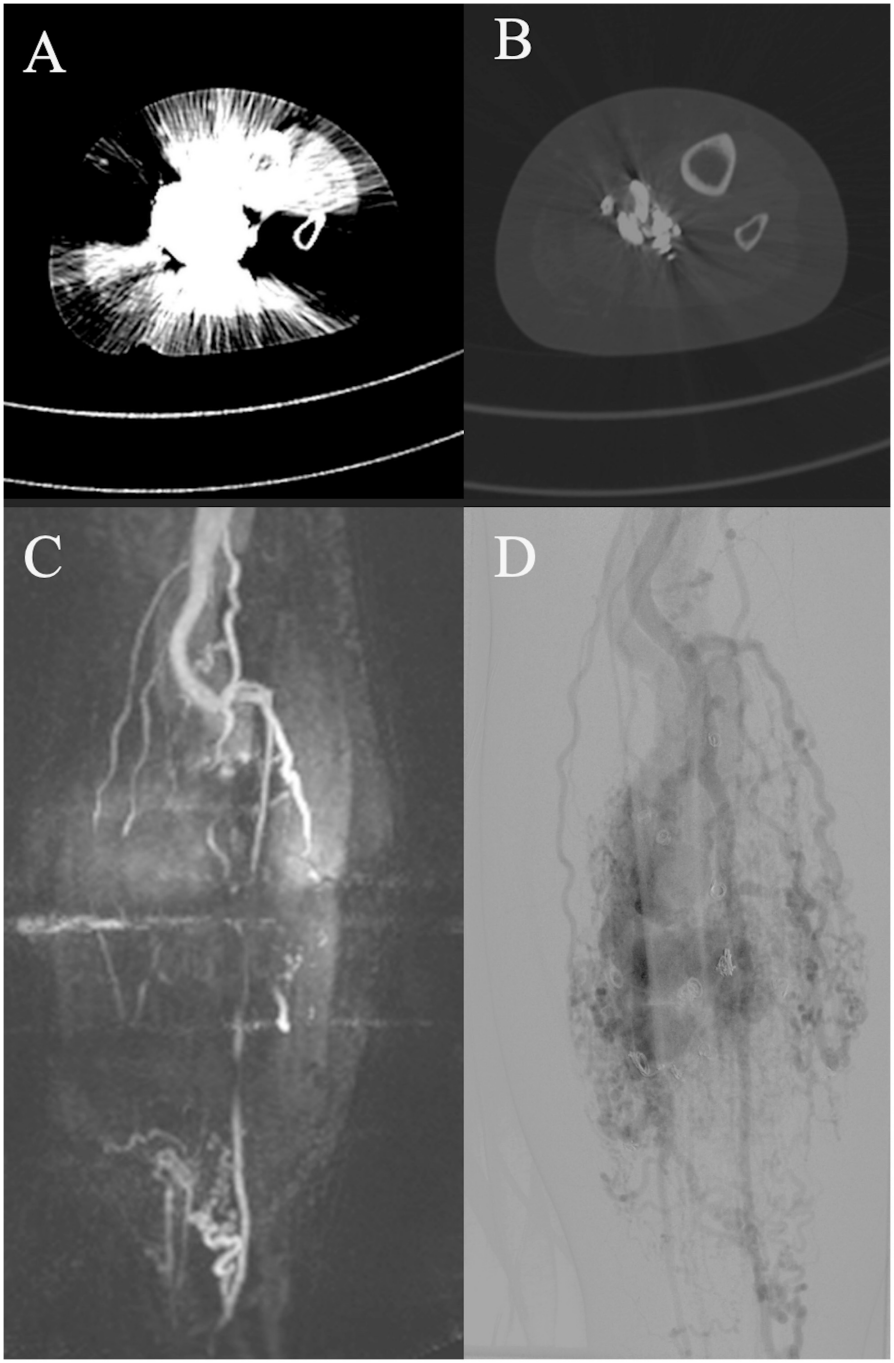
Diagnostic computed tomography (CT, **A, B**) and magnetic resonance tomography (MRI, **C**) of an adult patient with an arteriovenous malformation (AVM) of the left lower extremity after embolization with artifacts on CT and MRI. Pre-embolization digital subtraction angiography (DSA, **D**) shows an extensive AVM with dominant outflow veins and coil material after coil embolization before admission to the vascular anomaly center (VAC).

**Figure 7:**
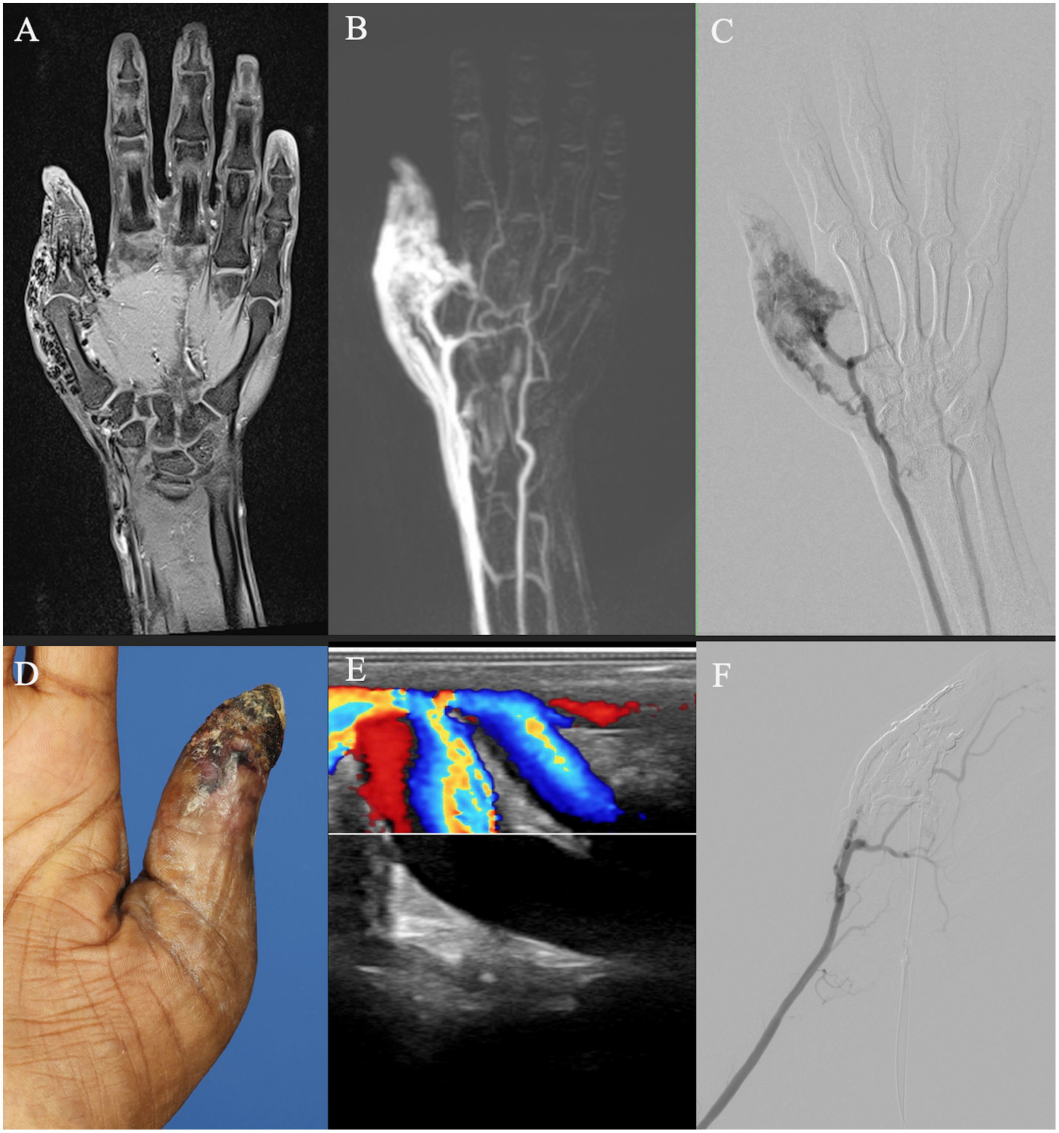
Imaging of an adult patient with an arteriovenous malformation of the right thumb. **(A, B)** Magnetic resonance imaging (MRI) shows a dilated vessel with flow voids on a T1 fat-saturated image after intravenous contrast agent admission **(A)**, and a vessel convolute involves the thumb area on a time-resolved angiography with interleaved stochastic trajectories (TWIST) MR angiography with already venous contrast due to the shunt volume **(B)**. **(C)** Digital subtraction angiography after arterial cubital puncture shows less arterial component than suspected on MRI. **(D)** Clinical photo of the patient's thumb. **(E)** Color Doppler ultrasound image demonstrates arterialized flow. **(F)** DSA after percutaneous embolization demonstrates devascularization of the AVM and earlier arterial perfusion of the palmar arch and the index finger.

Regarding diagnostic imaging, ultrasound was relevant for initial diagnostics and potentially during interventions of AVMs. However, unlike in slow-flow malformations, ultrasound is not suitable for evaluating the extent of the AVM or sufficient for therapy planning (25). MR-angiography, dynamic CT angiography, and angiography are important tools for treatment planning (10, 21, 26). When comparing MRI and CT regarding their provided information for therapy planning, MRI was more susceptible to motion artifacts, whereas CT, due to faster image acquisition, was less so. Both MRI and CT were limited in their diagnostic utility in previously treated patients due to artifacts from embolization materials. In these cases, catheter angiography provides better information regarding residual perfusion and available access routes to the NIDUS (Figure 6). Furthermore, both CT and MRI can be limited in assessing shunt dynamics and, consequently, in evaluating access routes to the NIDUS (transarterial, transvenous, and percutaneous) due to early shunting and therefore venous overlay (Figure 7). In our VAC, we decided to perform CT scans, especially in those patients who were unable to tolerate lying in the scanner for the duration of an MRI examination, either because of clinical impairment, claustrophobia, or refusal of sedation. In our analysis, three radiologists voted DSA as the best imaging modality to evaluate information on NIDUS localization and configuration. Thus, conventional angiography remains superior to MRI and CT for these evaluations. Because of its invasiveness, catheter angiography should be performed in cases where therapy is indicated, but not as a primary diagnostic tool (12). Many papers with descriptive information on diagnostic imaging in vascular malformations can be found (8, 10, 25, 27), but for extracranial AVMs, papers with the evaluation of imaging modalities regarding their provided information for treatment planning are rare.

The following limitations to this study must be considered: it is a retrospective database analysis, and over the past 10 years, multiple staff members have updated the database, which may have introduced potential inconsistencies. Furthermore, the relatively small patient size limits the statistical analyses. Additionally, a significant limitation concerns the diagnostic imaging prior to the initial patient's admission to our VAC. Many imaging studies were not documented, patients no longer had CDs with their images, or the images had been deleted from internal archives. Another limitation concerns the CT imaging evaluation we performed. There is an inconsistency in the way the CT scans were performed. Some scans were performed as dynamic or perfusion imaging with better NIDUS assessment, and others were monophasic or biphasic imaging studies, where dynamic behavior and therefore NIDUS assessment were limited.

## Conclusion

In summary, most patients with extracranial AVMs either present with or develop symptoms throughout their lives. The most common symptoms include pain, pulsation, and local hyperthermia. Additionally, motoric impairments are frequently observed, often due to pain and the size or location of the AVM. In our cohort, ulcerations and necrosis are uncommon but occur more frequently when the AVM is located in the hand. Right heart strain and eventual heart failure are quite rare.

Diagnostic imaging is essential for diagnosis and therapy planning. While the US is primarily relevant for confirming the diagnosis and cross-sectional imaging modalities are crucial for precise localization and depth assessment, our evaluation demonstrated that conventional angiography remains the superior method for therapy planning, access route determination, and NIDUS evaluation. Therefore, angiography should be performed prior to embolization procedures for treatment planning.

## Data Availability

The original contributions presented in the study are included in the article/supplementary material, further inquiries can be directed to the corresponding author.

## Footnotes

^1^ISSVA Classification of Vascular Anomalies ©2018 International Society for the Study of Vascular Anomalies. Available at: http://issva.org/classification.
